# Gesunde Lebenserwartung: Ein kritischer Blick auf Nutzen und Potenziale des demographischen Gesundheitsindikators

**DOI:** 10.1007/s00103-024-03874-w

**Published:** 2024-04-24

**Authors:** Marc Luy

**Affiliations:** Vienna Institute of Demography, Österreichische Akademie der Wissenschaften, Dominikanerbastei 16, 1010 Wien, Österreich

**Keywords:** Gesundheit, Expansion-Kompression, Healthy Life Years, Global Activity Limitation Indicator, Differential Item Functioning, Health, Expansion-compression, Healthy life years, Global activity limitation indicator, Differential item functioning

## Abstract

Die demographische Alterung hat vielfältige gesellschaftliche Konsequenzen, deren Ausmaß wesentlich vom Gesundheitszustand der Bevölkerung abhängt. Um diesen analysieren und bewerten zu können, wurden spezielle Kennziffern entwickelt, die in diesem Beitrag mit dem Überbegriff „gesunde Lebenserwartung“ (GLE) bezeichnet werden. Die Herleitung der GLE ist zwar intuitiv und leicht nachvollziehbar. Allerdings verdeckt eine zu einfache Interpretation die große Komplexität, die der Erweiterung der Sterbetafel um die Gesundheitsdimension innewohnt. Diese macht die GLE im Vergleich zur klassischen Lebenserwartung (LE) extrem empfindlich gegenüber verschiedensten konzeptionellen und methodischen Aspekten. In dem Beitrag wird dies für 3 Aspekte genauer dargestellt: die zugrunde liegende Definition von Gesundheit, die Wahl der Datenquelle als Grundlage für die Schätzung des Gesundheitszustands und das Berichtsverhalten der Survey-Teilnehmer. Dabei zeigt sich, dass die Auswirkung auf die GLE enorm sein kann, was zu erheblichen Verzerrungen bei der Interpretation von Niveaus und Trends, aber auch bei der Analyse von Unterschieden zwischen Bevölkerungen führt. Nichtsdestotrotz ist die Erweiterung der klassischen LE zur GLE eine wichtige Errungenschaft, die nicht aufgegeben werden darf. Deswegen werden in dem Beitrag auch Möglichkeiten diskutiert, wie der GLE-Indikator robuster und zuverlässiger gemacht werden könnte. Bis dies erreicht ist, darf die hohe methodische Sensibilität der GLE aber nicht ignoriert werden, wenn man mit ihr den Gesundheitszustand von Bevölkerungen bewertet und sie als Grundlage für gesundheitspolitische Maßnahmen herangezogen wird.

## Abstract (en)

Demographic ageing yields many societal consequences that depend strongly on the health status of the population. Special indicators have been developed for tracking and assessing population health, which are referred to with the overarching term healthy life expectancy (HLE). The derivation of HLE is intuitive and easily comprehensible. However, an overly simplistic interpretation hides the extreme complexity inherent in adding the health dimension to the life table. This makes HLE compared to classic life expectancy (LE) extremely sensitive to certain conceptual and methodological features. In the article, this is presented in more detail for three aspects: the underlying definition of health, the choice of survey data as a basis for estimating health status, and the reporting behavior of survey participants. It is shown that the impact on HLE can be enormous, leading to considerable bias in the interpretation of levels and trends, but also in the analysis of differences between populations. Nevertheless, the extension of classical LE to HLE is an important achievement that must not be abandoned. Therefore, the article also discusses ways in which the HLE indicator could be made more robust and reliable. Until this is achieved, however, the high methodological sensitivity of HLE must not be ignored if it is used to assess the health status of populations and as a basis for health policy measures.

## Hintergrund

Die demographische Entwicklung der deutschen Gesellschaft ist durch die Alterung der Bevölkerung gekennzeichnet, also die absolute und relative Zunahme von älteren im Vergleich zu jüngeren Menschen [1–3]. Bevölkerungsvorausberechnungen verdeutlichen, dass auch in Zukunft ein Fortschreiten der demographischen Alterung stattfinden wird [4]. Die zahlenmäßige und prozentuelle Zunahme der älteren Bevölkerung hat bereits heute gesellschaftliche Konsequenzen von unmittelbarer Relevanz, darunter eine steigende Nachfrage nach sozialen Dienstleistungen sowie erhöhte Anforderungen an die Gesundheitsversorgung und die sozialen Sicherungssysteme. Das Ausmaß dieser Konsequenzen hängt wesentlich vom Gesundheitszustand der Bevölkerung ab [5–7]. Dementsprechend wurde die Förderung der Gesundheit von der Europäischen Union (EU) als integraler Bestandteil von „Europe 2020“ definiert, ihrer 10-jährigen Strategie für wirtschaftliches Wachstum im Zeitraum 2010 bis 2020 [8]. Im Rahmen dieses Programms wurde 2011 die Pilotinitiative „European Innovation Partnership on Active and Healthy Ageing“ (EIP on AHA) ins Leben gerufen. Ihr Hauptziel war es, die gesunden Lebensjahre in der EU bis 2020 um 2 Jahre zu erhöhen [9, 10].

Um die Fortschritte derartiger Gesundheitsprogramme im Hinblick auf die Erreichung ihrer Ziele zu bewerten, ist ein zuverlässiger Indikator erforderlich, mit dem sich das Niveau und die Entwicklung der Gesundheit der Bevölkerung beobachten lassen. Zu diesem Zweck wurden spezielle Kennziffern entwickelt, die im Folgenden mit dem Überbegriff „gesunde Lebenserwartung“ (GLE) bezeichnet werden. Nach dem allgemeinen Verständnis ist die GLE schlicht eine Erweiterung der klassischen durchschnittlichen Lebenserwartung (LE) um eine Dimension. Technisch gesehen ist dies korrekt, denn letztlich werden die gesamten Lebensjahre der Sterbetafelpopulation – welche die Grundlage der LE bilden – in 2 Qualitätsdimensionen unterteilt: Lebensjahre, die in guter Gesundheit verbracht werden, und solche, die mit gesundheitlicher Beeinträchtigung gelebt werden. Allerdings verdeckt diese einfache Interpretation der GLE die große Komplexität, die der Erweiterung der Sterbetafel um die Gesundheitsdimension innewohnt. Diese macht die GLE im Vergleich zur LE extrem empfindlich gegenüber verschiedensten konzeptionellen und methodischen Aspekten, was zu erheblichen Verzerrungen bei der Interpretation von Niveaus und Trends, aber auch bei der Analyse von Unterschieden zwischen Bevölkerungen führen kann (siehe auch [11]). Diese Aspekte der GLE-Schätzung lassen sich grob in 2 Bereiche unterteilen: den konzeptionellen Bereich, mit dem vor allem Aspekte der Messung von Gesundheit gemeint sind, und den technischen Bereich, der die methodischen Aspekte der Schätzung der GLE beschreibt.

Zum konzeptionellen Bereich gehört allen voran die Definition von Gesundheit, die im Vergleich zur Sterblichkeit multidimensional und nicht einfach messbar ist. Für die meisten Schätzungen der GLE wird der Gesundheitszustand von Personen durch Selbsteinschätzungen auf der Grundlage spezifischer Fragen in Survey-Erhebungen erfasst, was zwangsläufig zu mehrdeutigen Resultaten führt [12]. Dies betrifft zum einen die Wahl des Surveys als Grundlage für die Schätzung der GLE, zum anderen spielen hier aber auch das Berichtsverhalten der Survey-Teilnehmer und deren Verständnis von Gesundheit bzw. Gesundheitsproblemen eine Rolle. Darüber hinaus hat auch die gewählte Definition von guter Gesundheit einen Einfluss, da die meisten Survey-Fragen zum Gesundheitszustand nicht dichotom sind und somit unterschiedliche Grenzen zwischen guter und beeinträchtigter Gesundheit ermöglichen. Gerade bei nichtdichotomen Gesundheitsindikatoren macht es einen Unterschied, ob man die gesunden Lebensjahre analysiert oder die gesundheitlich beeinträchtigte Lebenserwartung (BLE).

Zum technischen Bereich der Schätzung der GLE gehören zum Beispiel die Wahl der zugrunde gelegten Sterbetafel (Periodentafel, Kohortentafel oder eine Mischform), der Umgang mit den in Surveys vorkommenden fehlenden Antworten (Missing Cases), das Verfahren für die Glättung der altersspezifischen Anteilswerte des Gesundheitszustands bzw. die Verwendung nicht geglätteter Werte sowie die Größe der verwendeten Altersgruppen. Schließlich können auch der Beginn des obersten offenen Altersintervalls (z. B. 75+, 80+, 90+) sowie die methodische Kombination von Sterblichkeit und Gesundheit auf Basis von Prävalenzwerten (Sullivan-Verfahren auf Grundlage von Querschnittsdaten) oder Inzidenzwerten (Multi-State-Verfahren auf Basis von Längsschnittdaten) den Wert für die GLE beeinflussen. Obwohl das Multi-State-Verfahren die methodisch überlegene Technik ist, basieren die meisten und am häufigsten verwendeten Werte der GLE auf Schätzungen mit der Methode von Sullivan [13]. Dies liegt vor allem in der Datenverfügbarkeit begründet.^1^

Der vorliegende Beitrag beschränkt sich auf die Prävalenz-basierte Schätzung der GLE mit dem Sullivan-Verfahren. Dabei stellt der konzeptionelle Bereich der GLE-Schätzung eine bedeutendere Quelle möglicher Verzerrungen dar als der rein technische (zu Letzterem siehe z. B. [14–17]). Zur konkreten Veranschaulichung des Ausmaßes der möglichen Verzerrungen werden in diesem Beitrag daher 3 konzeptionelle Aspekte der Schätzung der GLE mit konkreten empirischen Beispielen genauer dargestellt:

1. die zugrunde liegende Definition von Gesundheit, also die Wahl des Gesundheitsindikators,
2. die Wahl der Datenquelle als Grundlage für die Schätzung des Gesundheitszustands der Bevölkerung und
3. das Berichtsverhalten der Survey-Teilnehmer.

Der Beitrag beschränkt sich dabei auf die 3 Gesundheitsindikatoren des sogenannten Minimum-European-Health-Moduls (MEHM), die den am häufigsten verwendeten Varianten der GLE zugrunde liegen und im folgenden Abschnitt näher vorgestellt werden. Bei der Darstellung der Effekte der methodischen Empfindlichkeiten des Indikators GLE stehen stets empirische Anwendungen für die deutsche Bevölkerung im Mittelpunkt. Ab dem zweiten Abschnitt zur Bedeutung der Datenquelle wird der Fokus auf die Europäische Union erweitert und auf den strukturellen Gesundheitsindikator der EU konzentriert, der als Healthy Life Years (HLY) bezeichnet wird. In der abschließenden Diskussion werden die wichtigsten Erkenntnisse noch einmal zusammengefasst und mögliche Ansätze zur Reduktion der methodischen Sensitivität der GLE diskutiert, die dabei helfen könnten, das Risiko von Fehlinterpretationen zu verringern.

## Definition von Gesundheit

In diesem Abschnitt geht es um die Bedeutung der Wahl des Gesundheitsindikators, die hier im Zusammenhang mit der für gesundheitspolitische Ziele wohl wichtigsten Frage demonstriert wird, nämlich ob die durch die zunehmende LE gewonnenen Lebensjahre hauptsächlich in guter oder beeinträchtigter Gesundheit verbracht werden. Diesbezüglich gibt es 3 theoretische Möglichkeiten [18]:

1. die gewonnenen Lebensjahre sind überwiegend solche, die in beeinträchtigter Gesundheit verbracht werden, das sogenannte Expansion-der-Morbidität-Szenario [19],
2. ein längeres Leben geht mit einer Verschiebung des Auftretens von Gesundheitsproblemen in höhere Alter einher, das sogenannte Kompression-der-Morbidität-Szenario [20], und
3. es besteht ein konstant ausgewogenes Verhältnis zwischen Gesundheit und Langlebigkeit, das sogenannte dynamische Gleichgewicht [21].

Die bisherigen empirischen Belege stützen alle 3 Szenarien, je nachdem, welche Gesundheitsindikatoren betrachtet werden [22]. Dies wird im Folgenden für die 3 Gesundheitsindikatoren des MEHM dargestellt. Diese beziehen sich auf:

- den allgemeinen Gesundheitszustand,
- das Vorhandensein chronischer Krankheiten und
- Einschränkungen bei normalen Alltagstätigkeiten.

Das MEHM wird in Deutschland seit 2005 jährlich in der auf Basis einer EU-Verordnung gesetzlich verankerten europäischen Gemeinschaftsstatistik „European Union Statistics on Income and Living Conditions“ (EU-SILC) erhoben [23]. Dabei wird bei Personen ab Alter 16 der allgemeine Gesundheitszustand erfasst mit der Frage: „Wie ist Ihr Gesundheitszustand im Allgemeinen?“, mit den Antwortmöglichkeiten: „sehr gut“, „gut“, „mittelmäßig“, „schlecht“ und „sehr schlecht“. Das Vorhandensein chronischer Krankheiten wird erhoben mit der Frage: „Haben Sie eine chronische Krankheit oder ein lang andauerndes gesundheitliches Problem?“, mit der Erläuterung: „Damit gemeint sind Krankheiten oder gesundheitliche Probleme, die mindestens 6 Monate andauern oder voraussichtlich andauern werden“, und den 2 Antwortmöglichkeiten: „Ja“ und „Nein“. Die Einschränkungen bei normalen Alltagstätigkeiten werden im MEHM mit einer einzigen Frage erfasst als Indikator für alle Arten von gesundheitlichen Einschränkungen, der als „Global Activity Limitation Indicator“ (GALI) bezeichnet wird. Die GALI-Frage lautet: „In welchem Ausmaß sind Sie durch Krankheit in der Ausübung Ihrer alltäglichen Arbeiten dauerhaft eingeschränkt?“, mit der Erläuterung: „Wir meinen damit seit mindestens einem halben Jahr“, und den 3 Antwortmöglichkeiten: „erheblich eingeschränkt“, „eingeschränkt, aber nicht erheblich“ und „nicht eingeschränkt“.

**Figure 1:**
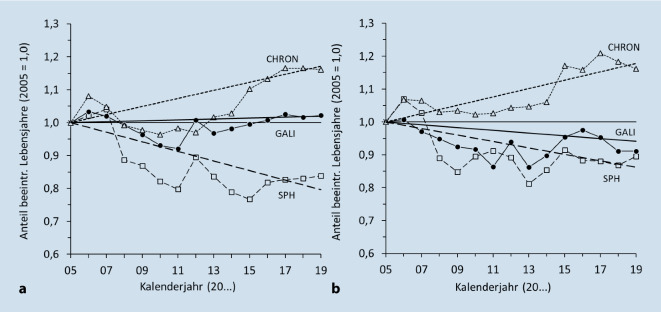

Um die Entwicklungen der GLE (ab Alter 16) den 3 theoretischen Trendszenarien der „Kompression-versus-Expansion-der-Morbidität“-Frage zuzuordnen, betrachten wir hier den Anteil der Lebensjahre an der gesamten LE, der in schlechter oder sehr schlechter Allgemeingesundheit, mit chronischen Krankheiten bzw. mit solchen gesundheitlichen Einschränkungen verbracht wird, die bei der GALI-Frage als „erheblich“ eingestuft sind. Die Ergebnisse für die BLE sind in Abb. 1a, b für Frauen und Männer dargestellt, wobei die Trends zur besseren Vergleichbarkeit auf den jeweiligen Ausgangswert für das Jahr 2005 normiert sind (im Jahr 2005 variiert die BLE je nach Gesundheitsindikator bei Frauen um 19,2 Jahre und bei den Männern um 16,3 Jahre).^2^ Folglich beginnen alle Verläufe mit dem Wert 1,0 und zeigen für die Folgejahre, wie sich der Anteil der gesundheitlich beeinträchtigten Lebensjahre in Bezug auf das Ausgangsjahr verändert hat. Dies gilt auch für die zusätzlich eingefügten linearen Trendverläufe.

Tatsächlich deuten sowohl bei den Frauen als auch bei den Männern die Trends in der BLE auf ganz unterschiedliche Entwicklungen hin. Bei der Allgemeingesundheit zeigt sich eine Reduktion des Anteils der beeinträchtigten Jahre, also eine Kompression der Morbidität, während sich bei den chronischen Krankheiten eine Expansion der Morbidität erkennen lässt. Die schwer interpretierbare Entwicklung der gesundheitlich eingeschränkten Lebensjahre entspricht am ehesten dem Szenario des dynamischen Gleichgewichts, da sich der Trend zwischen 2005 und 2019 – vor allem bei den Frauen – nahe am Wert 1,0 bewegt. Bei den Männern zeigt der Trend eher eine Tendenz in Richtung Kompressionsszenario, wenngleich deutlich weniger ausgeprägt, als dies bei der Allgemeingesundheit zu beobachten ist. Zusammengefasst lässt sich also sagen, dass jeder der Gesundheitsindikatoren des MEHM eine andere Antwort auf die Kompressions-Expansions-Frage für die deutsche Bevölkerung erbringt.

## Information zu Gesundheit aus Survey-Daten

Neben der Wahl des Gesundheitsindikators kann auch die Wahl des Surveys, aus dessen Daten die Information über den Gesundheitszustand der Bevölkerung abgeleitet wird, eine große Rolle für die Bestimmung der GLE spielen. Die Datengrundlage für die Bestimmung der altersspezifischen Prävalenzwerte für den Gesundheitszustand spielt aber nicht nur bezüglich abweichender Resultate zwischen verschiedenen Surveys eine Rolle. Bei Analysen von zeitlichen Entwicklungen können auch Veränderungen in den Fragestellungen innerhalb eines Surveys zu erheblichen Schwankungen führen. Dies soll hier am Beispiel der HLY, also der Lebensjahre ohne Einschränkungen, aufgezeigt werden. Wie bereits erwähnt, kommt diesem Indikator auf Basis des GALI eine besondere Bedeutung zu, da er den offiziellen Gesundheitsindikator der EU darstellt.

Trotz dieser hohen Bedeutung wurde die GALI-Frage in der deutschen SILC-Erhebung mehrfach modifiziert [24]. Die gravierendste Änderung erfolgte im Jahr 2015, als der GALI in 3 Teilfragen untergliedert wurde. Bis zur aktuellen Erhebung werden die SILC-Probanden nun zuerst nur gefragt: „Sind Sie dauerhaft durch ein gesundheitliches Problem bei Tätigkeiten des normalen Alltagslebens eingeschränkt?“, mit den beiden Antwortmöglichkeiten: „Ja“ und „Nein“. Nur im Fall der Ja-Antwort kommen dann die beiden Folgefragen: „Wie stark sind Sie bei Tätigkeiten des normalen Alltagslebens eingeschränkt?“, mit den beiden Antwortmöglichkeiten: „stark eingeschränkt“ sowie „mäßig eingeschränkt“, und schließlich: „Wie lange dauern Ihre Einschränkungen bereits an?“, mit den beiden Möglichkeiten: „weniger als 6 Monate“ und „6 Monate oder länger“.

**Figure 2:**
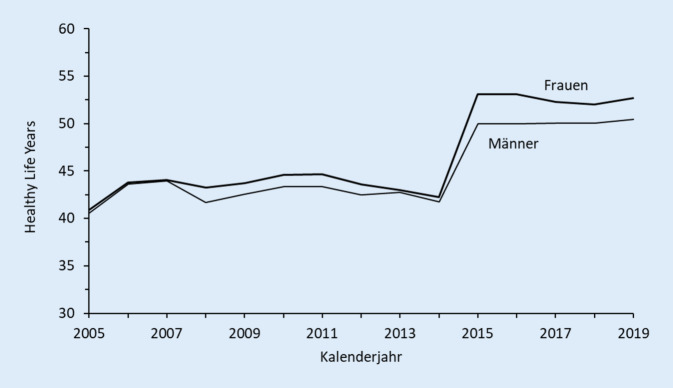

Abb. 2 verdeutlicht den Einfluss, den diese Änderung der GALI-Frage im Jahr 2015 auf den Trend der HLY für Deutschland ausübt. Innerhalb eines Jahres stiegen die HLY im Alter 16 um 10,8 Jahre bei den Frauen und um 8,3 Jahre bei den Männern. Eine Zeitreihenanalyse oder ein Vergleich von Kalenderjahren vor und nach dieser Modifikation im deutschen SILC ist dadurch schlichtweg nicht möglich. Die daraus resultierende Problematik wird besonders deutlich, wenn man sich die offizielle HLY-Statistik der EU im Licht der Zielsetzung der Europe-2020-Strategie vor Augen hält. Demnach stieg der Wert für die HLY, der von der EU unter bestimmten Annahmen bezüglich der Gesundheit in den im SILC nicht erfassten Altersstufen 0–15 für die gesamte Lebensspanne ab Geburt berechnet wird, für die deutsche Gesamtbevölkerung (Frauen und Männer zusammen) im Jahr 2015 um 10 Jahre an. Durch diesen enormen Anstieg der gesunden Lebensjahre erhöhten sich nach den Daten von Eurostat [25] auch die HLY für die Gesamtheit der 27 EU-Mitgliedstaaten (EU-27) – aufgrund der relativen Größe der deutschen Bevölkerung – im Vergleich zu 2014 um 1,5 Jahre. Auf diese Weise verhalf Deutschland der EU wesentlich zum Erreichen des Europe-2020-Ziels einer Erhöhung der durchschnittlichen HLY um 2 Jahre zwischen 2010 und 2020. Diese stiegen nämlich für die heutigen EU-27-Staaten innerhalb dieses 10-jährigen Zeitraums von 61,8 auf 64,0 Jahre, also um 2,2 Jahre, an. Dem eigentlichen und äußert wichtigen Sinn dieses ambitionierten Programms wird diese Statistik dadurch natürlich nicht gerecht.^3^

## Berichtsverhalten der Survey-Teilnehmer

In diesem Abschnitt soll schließlich noch ein Blick auf eine weitere Quelle potenzieller Verzerrung geworfen werden, die aus der Erfassung des Gesundheitszustands der Bevölkerung aus Survey-Fragen zur selbst eingeschätzten Gesundheit resultiert. Bereits frühere Studien haben gezeigt, dass die subjektive Einschätzung des Gesundheitszustands durch individuelle, situative und kulturelle Faktoren beeinflusst sein kann [26, 27]. Dazu gehören auch Unterschiede im Berichtsverhalten zwischen Bevölkerungen oder Bevölkerungsgruppen, was mit dem Fachbegriff „Differential Item Functioning“ (DIF) bezeichnet wird [28]. Um den Einfluss des Berichtsverhaltens auf internationale Vergleiche im HLY-Indikator abzuschätzen, entwickelten Luy et al. [29] ein Verfahren zum Ausgleich des DIF-Effekts. Dafür nutzen sie die in der ersten Welle des Survey of Health, Ageing and Retirement in Europe (SHARE) enthaltenen Gesundheitsvignetten [30] zur Quantifizierung der Heterogenität im Berichtsverhalten der Bevölkerungen aus 8 EU-Ländern. So wurden DIF-Korrekturfaktoren abgeleitet, mit denen die GALI-Prävalenzwerte aus der SILC-Erhebung angepasst wurden, um die Heterogenität im länderspezifischen Berichtsverhalten auszugleichen (für Details zu den Gesundheitsvignetten des SHARE siehe weiterer Beitrag von Luy in diesem Themenheft).

**Figure 3:**
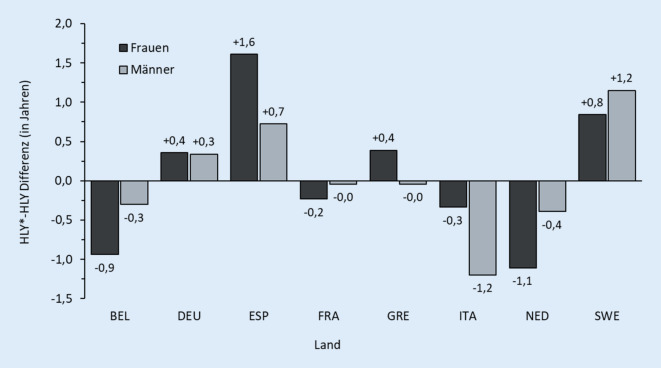

Abb. 3 zeigt für Frauen (dunkelgraue Balken) und Männer (hellgraue Balken) der 8 Länder die Differenzen zwischen DIF-angepasster HLY* und konventioneller HLY, wie sie für die jährlichen EU-Statistiken berechnet wird. Die Werte beziehen sich auf die verbleibenden Lebensjahre im Alter 50, dem Mindestalter der SHARE-Erhebung, und das Kalenderjahr 2005. Wie die Grafik zeigt, bewegt sich das Ausmaß der DIF-Anpassung in den HLY-Werten zwischen einer Reduktion um 1,2 Jahre bei Männern in Italien und einer Erhöhung der HLY um 1,6 Jahre bei Frauen in Spanien. Wie bei Luy et al. [29] im Detail beschrieben, führt die DIF-Anpassung auch in der Rangliste der 8 Länder zu teilweise erheblichen Verschiebungen. Bei den Frauen rücken Spanien um 3 Plätze und Schweden um einen Platz nach oben. Dagegen rutschen Belgien, Frankreich, Italien und die Niederlande um jeweils eine Position ab. Bei den Männern gewinnen Schweden 3 und Spanien 2 Plätze im Länder-Ranking, während Italien und die Niederlande 3 Ranglistenpositionen verlieren.^4^

Auch wenn die Ergebnisse der Studie von Luy et al. [29] aufgrund der zahlreichen erforderlichen Kompromisse und Approximationen bei der DIF-Anpassung der HLY nur als grobe Schätzungen verstanden werden dürfen, deuten sie nichtsdestotrotz darauf hin, dass eine durch kulturelle, soziale, normative oder linguistische Faktoren erzeugte Heterogenität im Berichtsverhalten zu Verzerrungen bei Vergleichen von Daten zur selbst eingeschätzten Gesundheit zwischen Bevölkerungen führen kann. Hervorzuheben ist vor allem die Konsistenz in der Richtung der Korrektur der HLY-Werte bei Männern und Frauen, die – wie in der Studie von Luy et al. [29] gezeigt – sogar über die Altersgruppen bestehen bleibt.^5^ Diese Konsistenz ist ein starker Indikator dafür, dass es tatsächlich länderspezifische Einflussfaktoren auf das Berichtsverhalten der Survey-Teilnehmer gibt. Vor allem die unterschiedlichen Sprachen können hier eine einflussreiche Rolle spielen. Bei den großen internationalen Survey-Erhebungen werden die Fragebögen nämlich in die jeweiligen Landessprachen übersetzt, was zu einem unterschiedlichen Verständnis der in einer bestimmten Frage beschriebenen Gesundheitsmerkmale führen kann. Dies könnte sich schließlich auf das Ausmaß der beschriebenen Gesundheitsprobleme auswirken, die im oben beschriebenen DIF-Effekt zum Ausdruck kommen.

## Diskussion

Die GLE wird generell als robuster Indikator verstanden, der in jüngster Vergangenheit die klassische LE als zentrale Maßzahl für den Gesundheitszustand einer Bevölkerung abgelöst hat. Dabei wird jedoch meist übersehen, dass die GLE wesentlich stärker durch eine Vielzahl technischer und konzeptioneller Faktoren beeinflusst werden kann als die klassische LE, die ihrerseits schon weniger klar zu interpretieren ist als gemeinhin angenommen [31]. Die in diesem Beitrag demonstrierten Beispiele zeigen, dass die Statistiken zum Niveau und zu den Trends der GLE je nach den zugrunde liegenden Erhebungsdaten und Gesundheitsindikatoren zum Teil erheblich variieren (siehe auch [32, 33]). Die Nichtberücksichtigung der dadurch möglichen Verzerrungen bei Vergleichen von GLE-Werten zwischen verschiedenen Zeitpunkten oder Bevölkerungen beinhaltet das Risiko von Fehleinschätzungen, die dann auch Eingang in bevölkerungspolitische Entscheidungen haben können.

Im ersten Abschnitt des Beitrags wurde gezeigt, dass die 3 MEHM-Indikatoren für den Gesundheitszustand eine unterschiedliche Entwicklung des Gesundheitszustands der deutschen Bevölkerung signalisieren: Kompression der Morbidität bei der Allgemeingesundheit, Expansion der Morbidität bei den chronischen Krankheiten und ein dynamisches Gleichgewicht zwischen der mit Alltagseinschränkungen verbrachten Lebenszeit und den gesamten Lebensjahren. Dies wirft die Frage auf, warum die verschiedenen Gesundheitsindikatoren zu unterschiedlichen Schlussfolgerungen führen. Ein wesentlicher Unterschied zwischen den 3 Gesundheitsdimensionen des MEHM liegt in der Schwere ihrer Krankheitsverläufe, die sich in ihrem Zusammenhang mit dem Sterberisiko widerspiegelt. Analysen mit Längsschnittdaten für Deutschland haben gezeigt, dass das Sterberisiko bei Personen mit schlechter Allgemeingesundheit und Einschränkungen bei Alltagstätigkeiten deutlich höher ist als bei Personen, die angeben, an einer oder mehreren chronischen Krankheiten zu leiden [34]. Vor diesem Hintergrund machen die unterschiedlichen Trends in der GLE durchaus Sinn. Der Anstieg in der gesamten LE muss seinen Ursprung ja in der Reduktion von Krankheiten mit hohem Sterberisiko haben, was den Kompressionseffekt bei der Allgemeingesundheit erklären kann. Chronische Krankheiten führen dagegen weniger häufig bzw. später zum Tod. Dafür steigt die Wahrscheinlichkeit, an einer chronischen Krankheit zu leiden, mit dem Alter an. Somit lässt sich schlussfolgern, dass die Kompression der Morbidität bei der Allgemeingesundheit eine Ursache für den Anstieg der durchschnittlichen LE ist, während die Expansion der Morbidität bei den chronischen Krankheiten eine Folge des Anstiegs der LE ist (siehe auch [24]).

Die unterschiedlichen Trends der GLE auf Basis der 3 MEHM-Indikatoren verdeutlichen, dass der aus der Gesundheitspolitik an die Wissenschaft herangetragene Wunsch, den Gesundheitszustand der Bevölkerung mit einer einzigen Maßzahl zu beschreiben, aufgrund der Komplexität der Gesundheit mit ihren vielen verschiedenen Ausprägungen und Formen der Beeinträchtigung kaum zu erfüllen ist. Eine mögliche Lösung könnte in der Kombination verschiedener Gesundheitsindikatoren liegen. Lazarevič [35] fasste mit Daten des deutschen Alterssurveys der Jahre 2008 und 2014 die 3 Dimensionen des MEHM zu einem generischen Gesundheitsindikator zusammen und beurteilte seine Nützlichkeit im Vergleich zur selbst eingeschätzten Allgemeingesundheit. Seine Analysen zeigen, dass der generische MEHM-Indikator eine gute interne Konsistenz aufweist und eine eigene latente Variable darstellt. Allerdings kann der von Lazarevič [35] entwickelte Ansatz in der bisherigen Form nicht für die Schätzung der GLE verwendet werden. Weiterentwicklungen der Methodik in diese Richtung wären daher wertvoll.

Ein alternativer Ansatz, die methodische Sensibilität der GLE zu reduzieren, wurde von Muszyńska-Spielauer und Luy [36] vorgeschlagen. Dieser besteht darin, die von den Survey-Teilnehmern angegebenen Schweregrade der gesundheitlichen Probleme mit der damit verbundenen Reduktion in der allgemeinen Lebenszufriedenheit zu gewichten. Der als „Well-being Adjusted Health Expectancy“ (WAHE) bezeichnete Indikator wurde hier ebenfalls für die 3 MEHM-Indikatoren getestet, sowohl einzeln für die 3 Gesundheitsdimensionen als auch kombiniert für alle zusammen. Die Resultate zeigen, dass WAHE sowohl in der ein- also auch in der multidimensionalen Variante robuste Ergebnisse liefert, die weniger stark vom gewählten Gesundheitsindikator abhängen als die konventionellen GLE-Indikatoren.

Obwohl die Ergebnisse der beiden Studien vielsprechende Möglichkeiten aufzeigen, wie das Problem der Multidimensionalität der Gesundheit für die Bestimmung eines globalen Gesundheitsindikators reduziert werden könnte, können auch durch sie die in den beiden anderen Abschnitten des Beitrags dargestellten Verzerrungsfaktoren der Survey-Daten und des Berichtsverhaltens der Survey-Teilnehmer nicht eliminiert werden. Vor allem der im zweiten Abschnitt gezeigte Effekt der Modifikation der GALI-Frage in der deutschen SILC-Erhebung auf die Entwicklung der von gesundheitlichen Einschränkungen bei Alltagstätigkeiten freien Lebensjahre, dem offiziellen HLY-Indikator der EU, verdeutlicht die große Gefahr der Verzerrung von GLE-Statistiken durch methodisch-technische Artefakte.

## Fazit

Zusammengefasst legen die in diesem Beitrag demonstrierten methodischen und konzeptionellen Empfindlichkeiten der gesunden Lebenserwartung (GLE) die Schlussfolgerung nahe, dass sie ihrer großen Bedeutung als zuverlässiger Indikator, mit dem sich das Niveau und die Entwicklung der Gesundheit der Bevölkerung verfolgen lässt, nicht gerecht wird. Zumindest bezüglich des Healthy Life Years-Indikators der Europäischen Union könnte eine Verbesserung der Situation vielleicht dadurch erreicht werden, dass die Frage des zugrunde liegenden Global Activity Limitation Indicator (GALI) vereinfacht und die Gesundheitsausprägungen dichotomisiert werden, z. B. in der Form: „Sind Sie durch ein gesundheitliches Problem in der Ausübung Ihrer Alltagstätigkeiten eingeschränkt?“, mit den beiden einzigen Antwortmöglichkeiten: „Ja“ und „Nein“. Dadurch würden vor allem die Modifikationsmöglichkeiten in Fragestellung und Antwortvorgaben deutlich reduziert. Auch der Effekt eines zwischen den Landessprachen unterschiedlichen Verständnisses der abgefragten Gesundheitsprobleme könnte so vermutlich minimiert, wenngleich wohl nicht völlig eliminiert werden.

Bei all den in diesem Beitrag formulierten Kritikpunkten gegen den GLE-Indikator darf aber nicht vergessen werden, dass es sich hier um eine noch sehr junge methodische Entwicklung der demographischen Gesundheitsforschung handelt. Deshalb ist es verständlich, dass viele methodische Detailfragen noch nicht beantwortet und manche Aspekte der Empfindlichkeiten dieses speziellen Indikators noch nicht in den Fokus der Forschung gerückt sind. Der mit der Erweiterung der klassischen Lebenserwartung zur GLE eingeschlagene Weg ist eine wichtige Errungenschaft, die nicht aufgegeben werden darf. Schließlich dürfte für die meisten Menschen die Anzahl der gesunden Lebensjahre wesentlich bedeutsamer sein als die gesamte Anzahl an Lebensjahren. Bis jedoch eine möglichst robuste Variante der GLE gefunden und allgemein akzeptiert ist, dürfen die zum Teil großen Empfindlichkeiten der bislang existierenden GLE-Varianten nicht ignoriert werden, wenn mit ihnen der Gesundheitszustand von Bevölkerungen analysiert bzw. bewertet wird und sie als Grundlage für gesundheitspolitische Maßnahmen herangezogen werden.

## Footnotes

1 Für die Verwendung des Sullivan-Verfahrens genügen eine Querschnittserhebung des Gesundheitszustands sowie eine Perioden-Sterbetafel, wohingegen das Multi-State-Verfahren mehrere Längsschnitterhebungen von Gesundheitszustand und Sterblichkeit der Survey-Teilnehmer erfordert.

2 Die höchsten Werte für die BLE im Alter 16 zeigen sich bei den chronischen Krankheiten. Für das Jahr 2005 liegen diese für Frauen bei 25,5 Jahren und für Männer bei 21,1 Jahren. Für die mit erheblichen Einschränkungen im Alltag verbrachten Lebensjahre beträgt die BLE im Jahr 2005 für Frauen 6,3 Jahre und für Männer 4,8 Jahre. Für die mit schlechter bzw. sehr schlechter Allgemeingesundheit verbrachten Lebensjahre ergeben sich für Frauen 6,8 Jahre und für Männer 5,4 Jahre.

3 Als Randnotiz kann noch hinzugefügt werden, dass Deutschland durch die Veränderung der GALI-Frage in der HLY-Rangliste der 27 EU-Mitgliedstaaten innerhalb eines Jahres von Platz 24 auf Platz 4 gesprungen ist.

4 Deutschland befindet sich unter den analysierten Daten für 2005 bei Frauen und Männern sowohl bei den konventionellen als auch bei den DIF-angepassten HLY-Werten am untersten Ende der Rangliste der 8 Länder. Dies ist auf die im vorangegangenen Abschnitt beschriebenen im Vergleich zu anderen westeuropäischen Ländern bis zur Modifikation der GALI-Frage im Jahr 2015 deutlich niedrigeren HLY-Werte zurückzuführen.

5 Die einzige Ausnahme ist Griechenland, wo die DIF-Anpassung bei den Männern zu einer Erhöhung und bei den Frauen zu einer Reduktion der HLY führt. Diese DIF-Anpassungen sind aber so geringfügig, dass man hier nicht wirklich von einer bei Frauen und Männern gegensätzlich gerichteten Korrektur sprechen kann.
